# Supplementary material for: DiMSum: an error model and pipeline for analyzing deep mutational scanning data and diagnosing common experimental pathologies
Source: Genome Biol. 2020 Aug 17;21:207. doi: 10.1186/s13059-020-02091-3 (PMC7429474; doi:10.1186/s13059-020-02091-3)
Supplement: Supplementary file 1 — Additional file 1. Supplementary Figures S1 to S10. [file 13059_2020_2091_MOESM1_ESM.pdf]

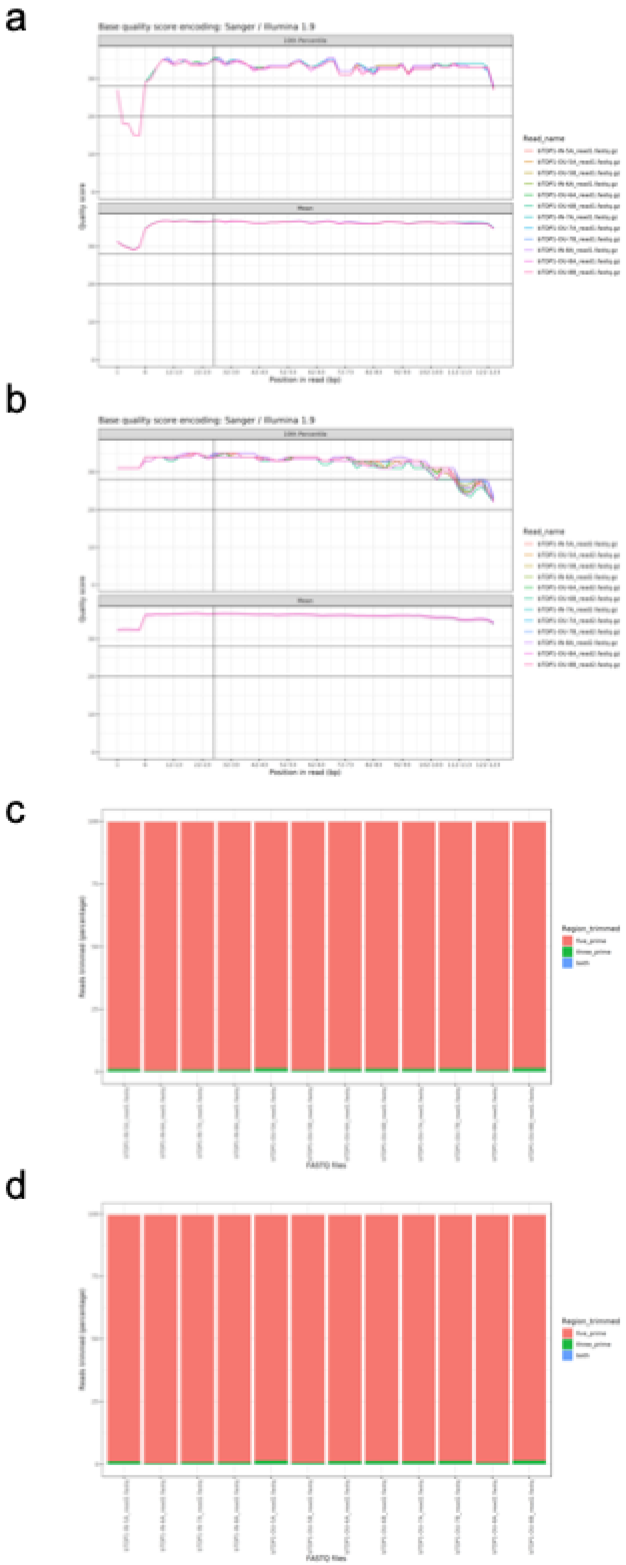

Figure S1. **DiMSum pipeline report: Raw FastQ file quality control summary and constant region trimming statistics. a.** 10th percentile (upper) and mean (lower) Phred quality scores

shown for forward reads (Read 1) in all FastQ files (see legend). **b**. Similar to A except quality scores for reverse reads (Read 2) are shown. Vertical dashed lines separate variable from constant regions. **c.** Percentage of forward reads (Read 1) in which corresponding 5' and/or 3' constant regions were matched and trimmed (see legend), shown separately for each FastQ file. **d.** Similar to A except trimming statistics are shown for reverse reads (Read 2).

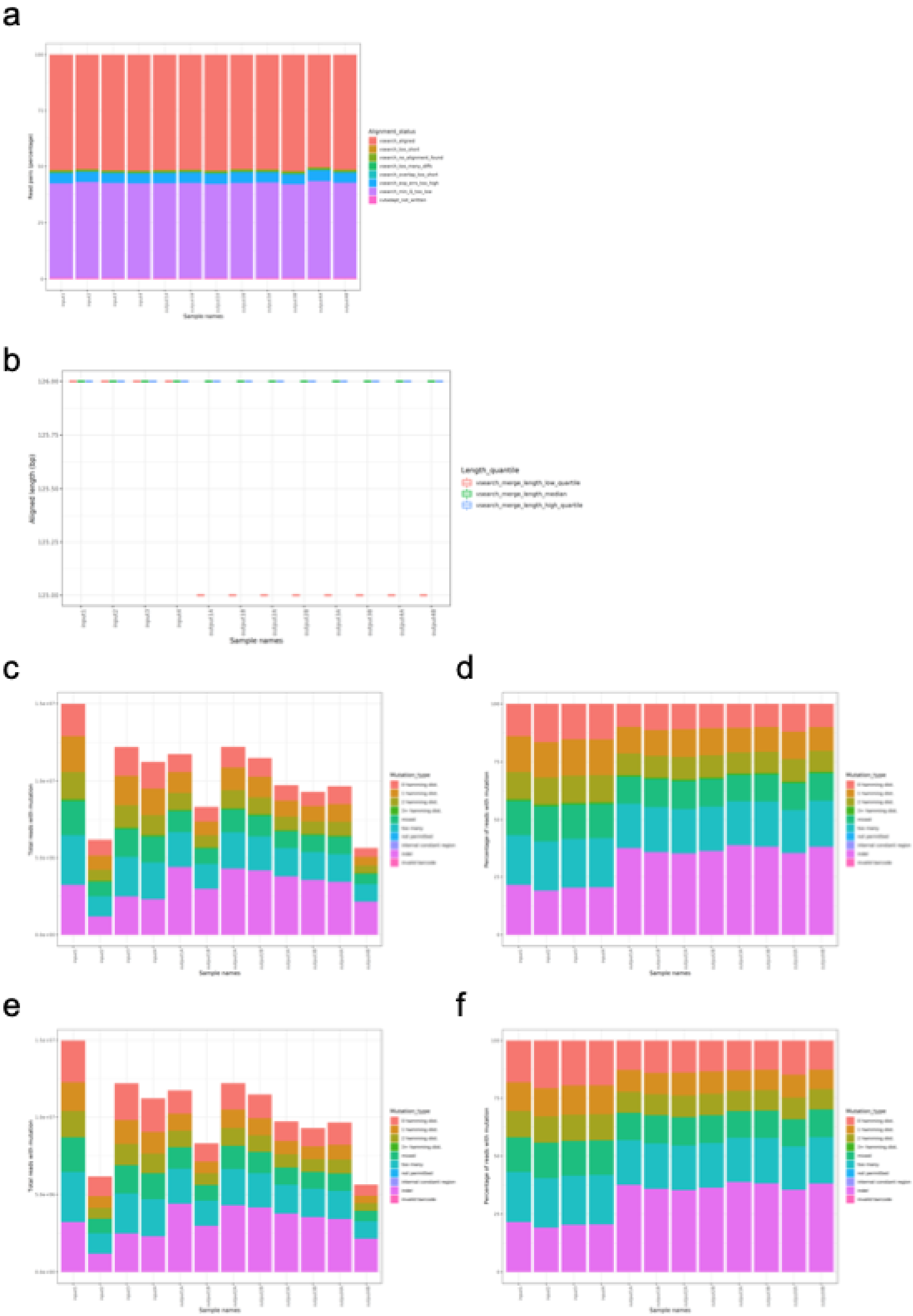

Figure S2. **DiMSum pipeline report: Paired-end read alignment statistics and variant processing and mutation statistics. a.** Percentage of successfully aligned paired-end reads

("vsearch_aligned") retained for downstream analysis. Remaining read pairs not matching user-specified criteria are discarded. **b**. Aligned read length distributions shown separately for all samples (see legend). **c.** Per-sample total counts (and percentages, **d**) of retained processed reads with 0,1,2 or >3 (3+) nucleotide substitutions (see legend), as well as those discarded due to "invalid barcodes" (in the case of barcoded libraries), "indel" mutations, mutated "internal constant regions", non-intended mutations ("not permitted"), variants with more substitutions than desired ("too many") and nonsynonymous variants that have synonymous substitutions in other codons ("mixed"). **e** and **f** are similar to a and b, but indicate total counts and per sample percentages of read amino acid mutation statistics respectively (shown if the target molecule was a coding sequence).

a

b

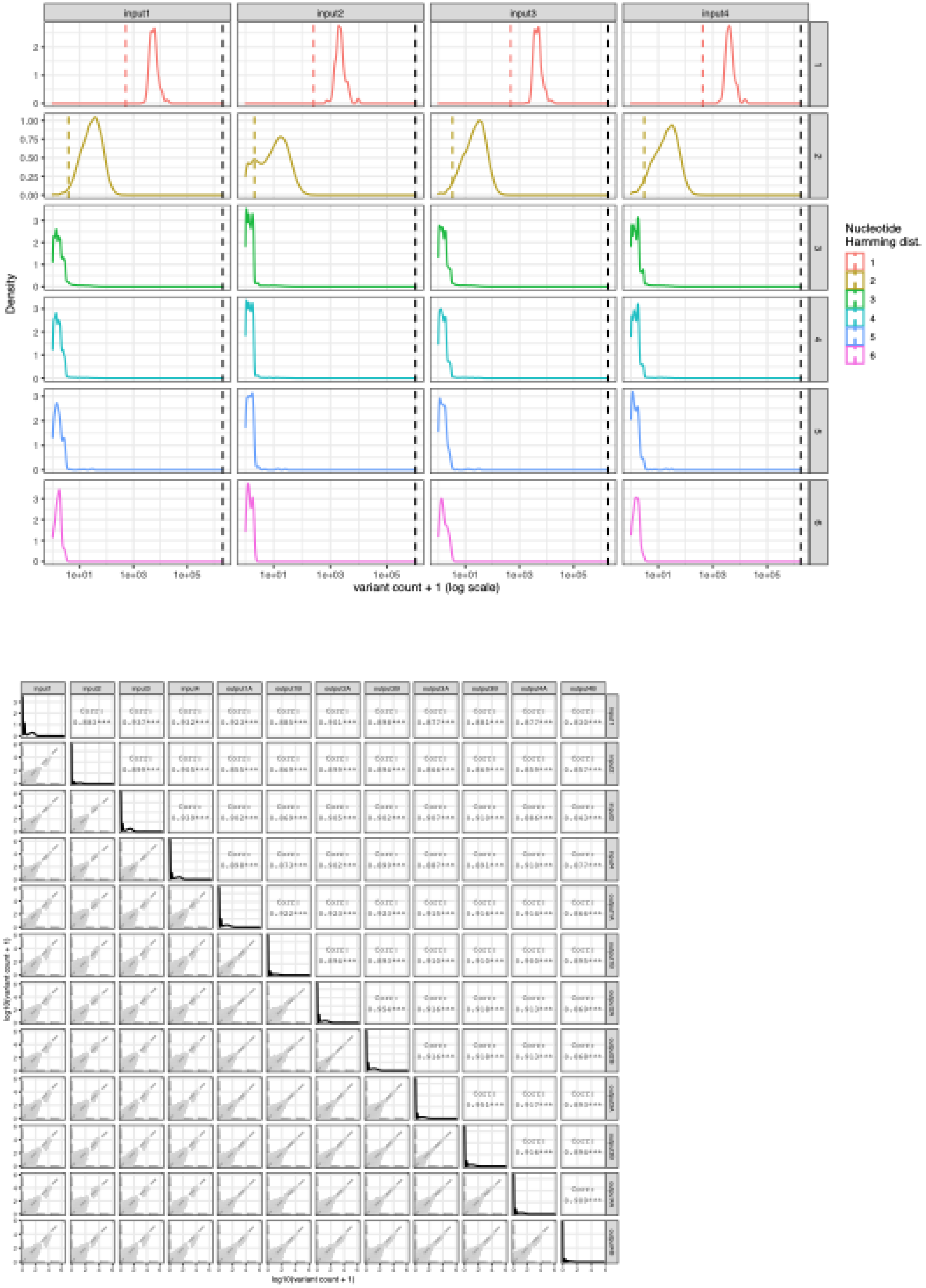

Figure S3. **DiMSum pipeline report: Marginal input variant count distributions and inter-sample variant count diagnostic plot. a.** Input sample variant count distributions for nucleotide substitution variants with hamming distances to the wild type sequence of up to 6.

Wild-type counts are indicated by the black dashed line. Expected “fictional” variant count frequencies are indicated by red and brown dashed lines for single and double nucleotide substitution variants respectively. Bimodal distributions and unimodal distributions not surpassing indicated thresholds are indicative of variants originating from sequencing errors due to a library bottleneck (see Figure 4a). **b**. Scatterplot matrix depicting correlation between all Input and Output sample variant counts. Matrix cells in the upper triangle show Pearson correlation coefficients. Matrix cells in the lower triangle show hexagonal heatmaps, where each hexagon is shaded by corresponding two dimensional bin counts. Matrix diagonal cells indicate count densities. Distinct variant populations or 'flaps' - subsets of variants that appear at high counts in one replicate but at low counts in another – not resulting from applied selection are indicative of replicate or DNA extraction bottlenecks (see Figure 4b).

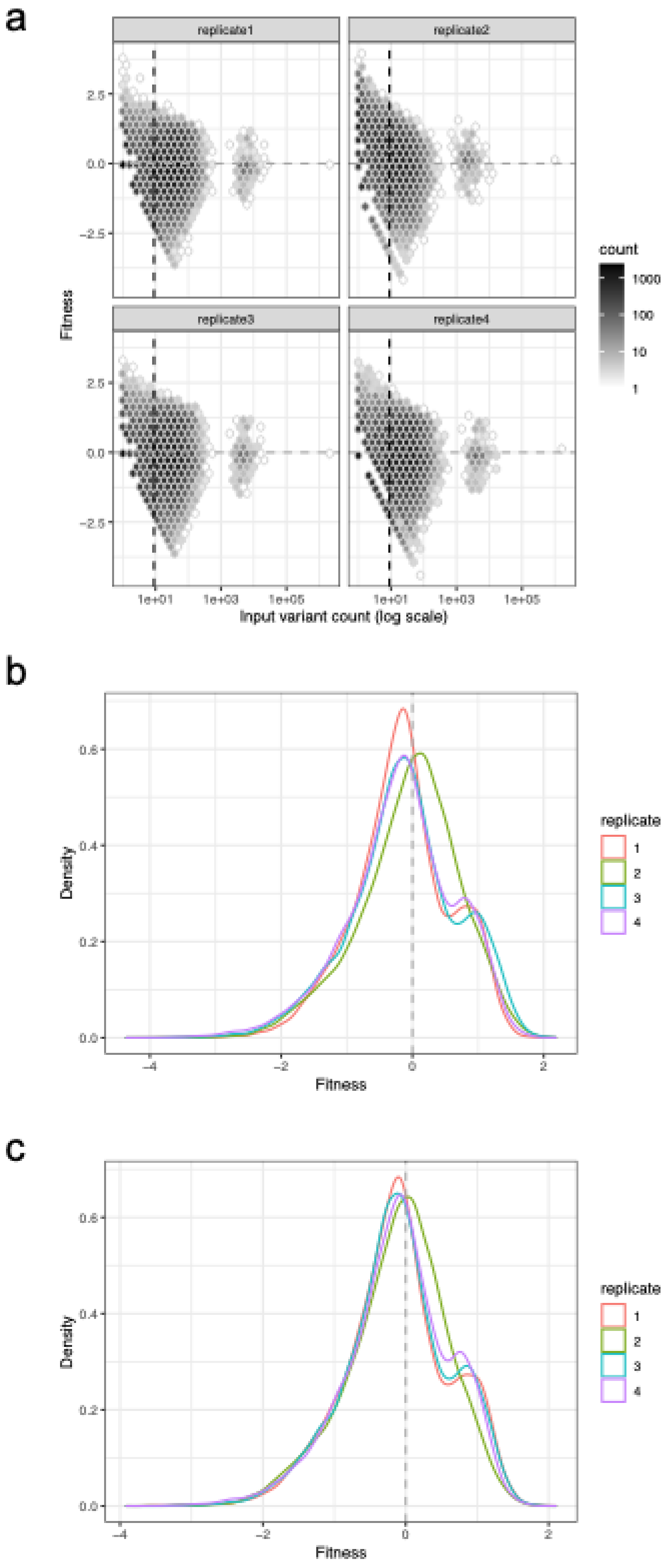

Figure S4. **DiMSum pipeline report: Input read threshold for full fitness range and fitness normalisation. a.** Two dimensional hexagonal heatmaps showing replicate fitness scores

versus Input variant counts. The vertical dashed line indicates the minimum Input count threshold used for retaining variants (covering the full fitness range) for subsequent error model fitting. **b**. Replicate fitness distributions before (and after, **c**) ‘scale and shift’ inter-replicate normalisation. Deviations in distribution shape (e.g. replicate 2) indicate systematic errors between replicates; affected replicates might have to be excluded from error model fitting and downstream analyses.

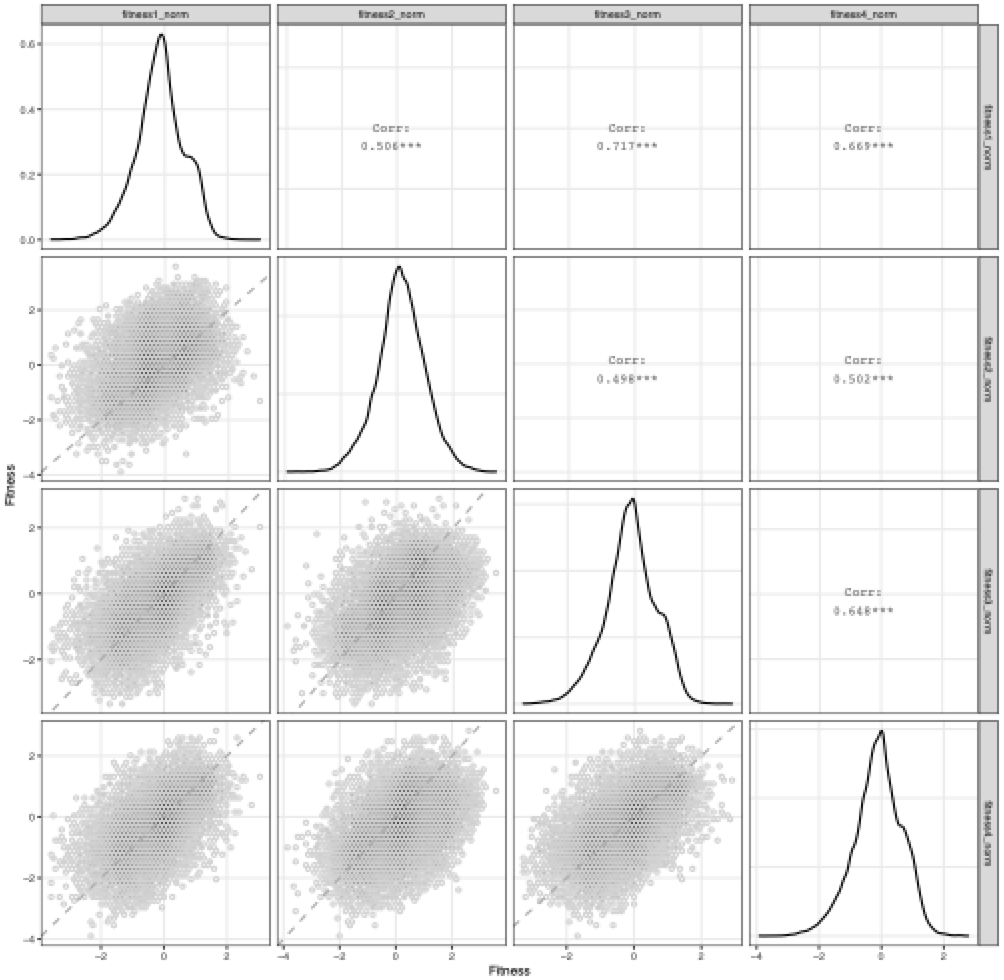

Figure S5. **DiMSum pipeline report: Inter-sample variant fitness diagnostic plot.** Two dimensional hexagonal heatmaps showing replicate fitness score correlations (cells in the lower matrix triangle), corresponding Pearson correlation (upper triangle) and marginal densities (matrix diagonal). Only variants retained during error model fitting are depicted.

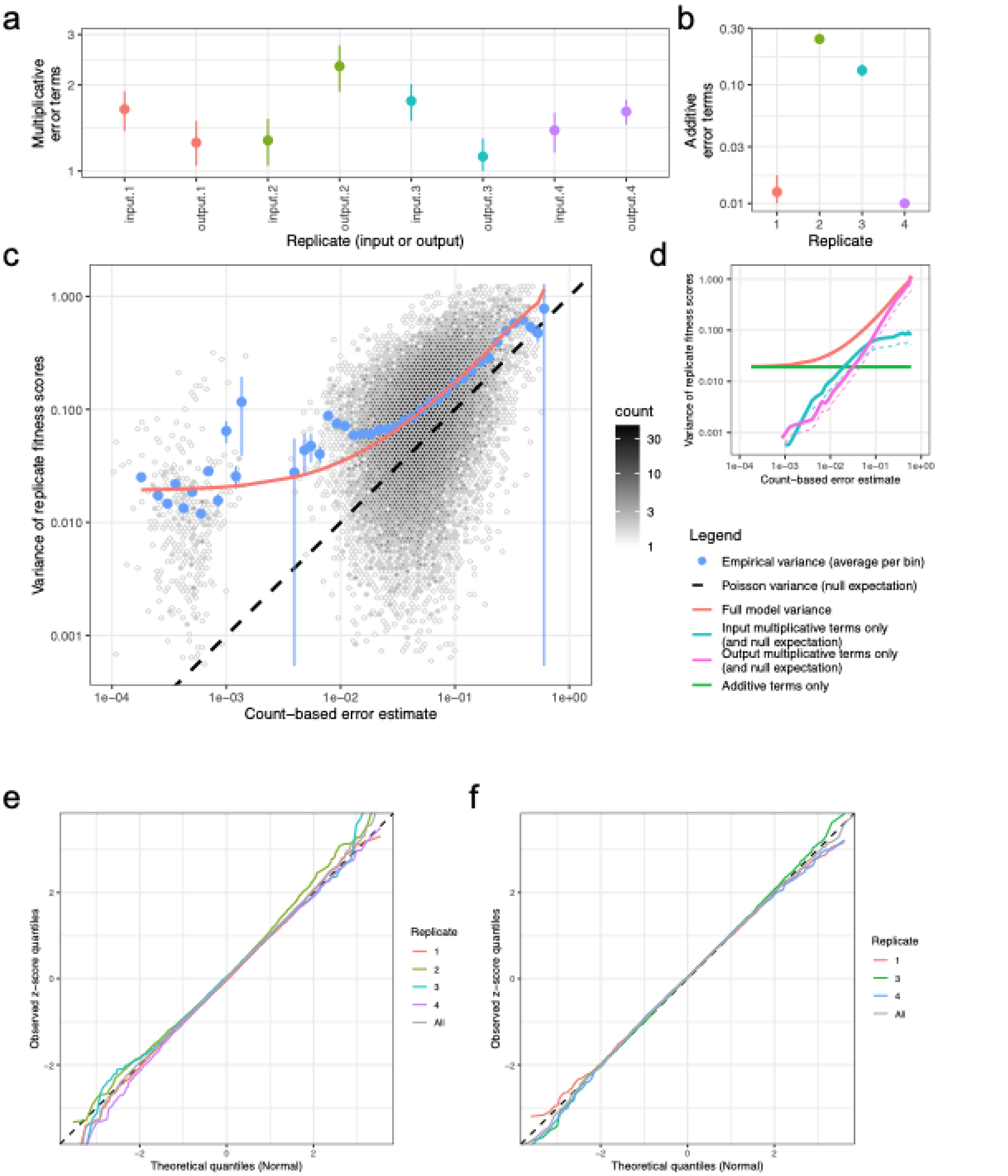

Figure S6. **DiMSum pipeline report: Fitness error model estimates. a**. and **b**. Multiplicative (panel a) and additive (panel b) error terms estimated by the error model on TDP-43 290-331 dataset. Dots give mean parameters, error bars 90% confidence intervals. **c**. Empirical variance of replicate fitness scores as a function of error estimates purely based on sequencing counts under Poisson assumptions. Empirical variance (blue dots show average variance in equally-spaced bins, error bars indicate avg. variance(1 + 2 / #variants per bin)) is

over-dispersed compared to baseline expectation of variance being described by a Poisson distribution. The bimodality of the count-based error distribution results from relatively few single nucleotide mutants with high counts (thus low count-based error) and many double nucleotide mutants with low counts (thus higher count-based error). The DiMSum error model (red line) accurately captures deviations of the empirical variance from Poisson expectation (black dashed line). **d**. Cyan and magenta lines indicate multiplicative error term contributions corresponding to Input and Output samples respectively. The horizontal green line indicates the additive error term contribution. The red line indicates the full DiMSum error model. **e, f**. DiMSum outputs quantile-quantile plots of z-scores of the differences between replicate fitness scores as a visual check whether the error model has properly estimated error magnitudes. Z-scores are shown for individual replicates (the indicated replicate is the 'test' replicate) and aggregated across all replicates (grey, as shown in Figure 3 for different error models). Shown here is the difference between DiMSum error models when replicate 2 is included in the analysis (panel **e**) or excluded from the analysis (panel **f**). In the first analysis, replicate two shows biased fitness scores, while the latter analysis the three remaining replicates have unbiased fitness scores with the correct error magnitude.

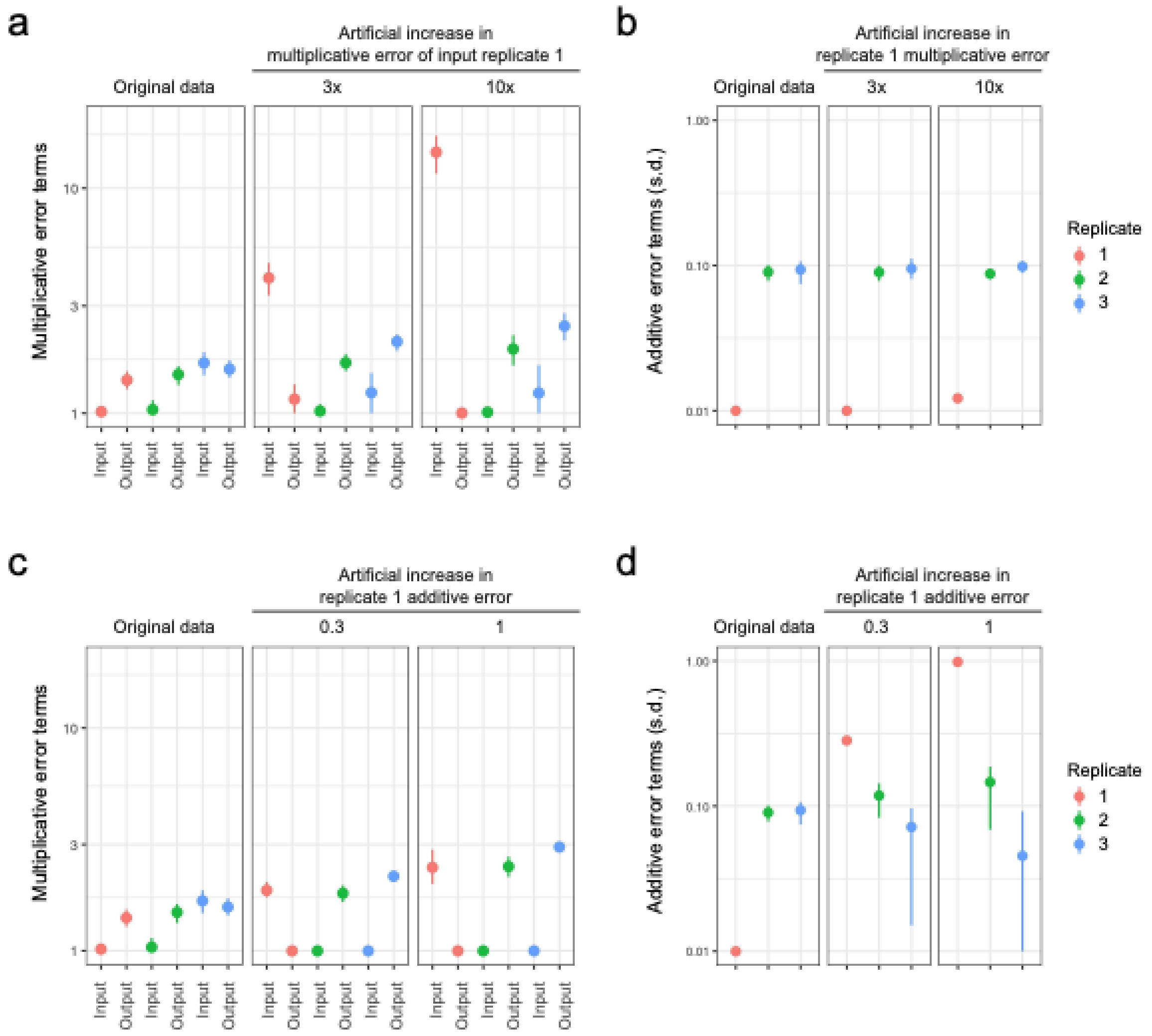

Figure S7. **DiMSum error model estimates multiplicative and additive error sources in fitness scores. a**. and **b**. Multiplicative (panel **a**) and additive (panel **b**) error terms estimated by the error model on TDP-43 290-331 dataset after multiplicative manipulation (middle and right panels) of the original data (left panels). **c**. and **d**. Multiplicative (panel **c**) and additive (panel **d**) error terms after additive manipulation (middle and right panels) of the original data (left panels). The DiMSum error model captures multiplicative (panel **a**) and additive (panel **d**) manipulations of the original dataset, with minor effects on non-manipulated parameters. Of note, manipulating replicate 1 input multiplicative error results in minor lowering of replicate 1 output multiplicative error, possibly due to slight overlap in variants that are similarly affected by both error terms. Additionally, at very high addition of replicate 1 additive error, multiplicative errors are affected. This is likely because at a magnitude of 1 (s.d.) the additive error dominates the overall error (and exceeds by far any additive error term observed in published DMS datasets), masking error contributions by other error sources and thus complicate their reliable estimation. Dots give mean parameters, error bars 90% confidence intervals.

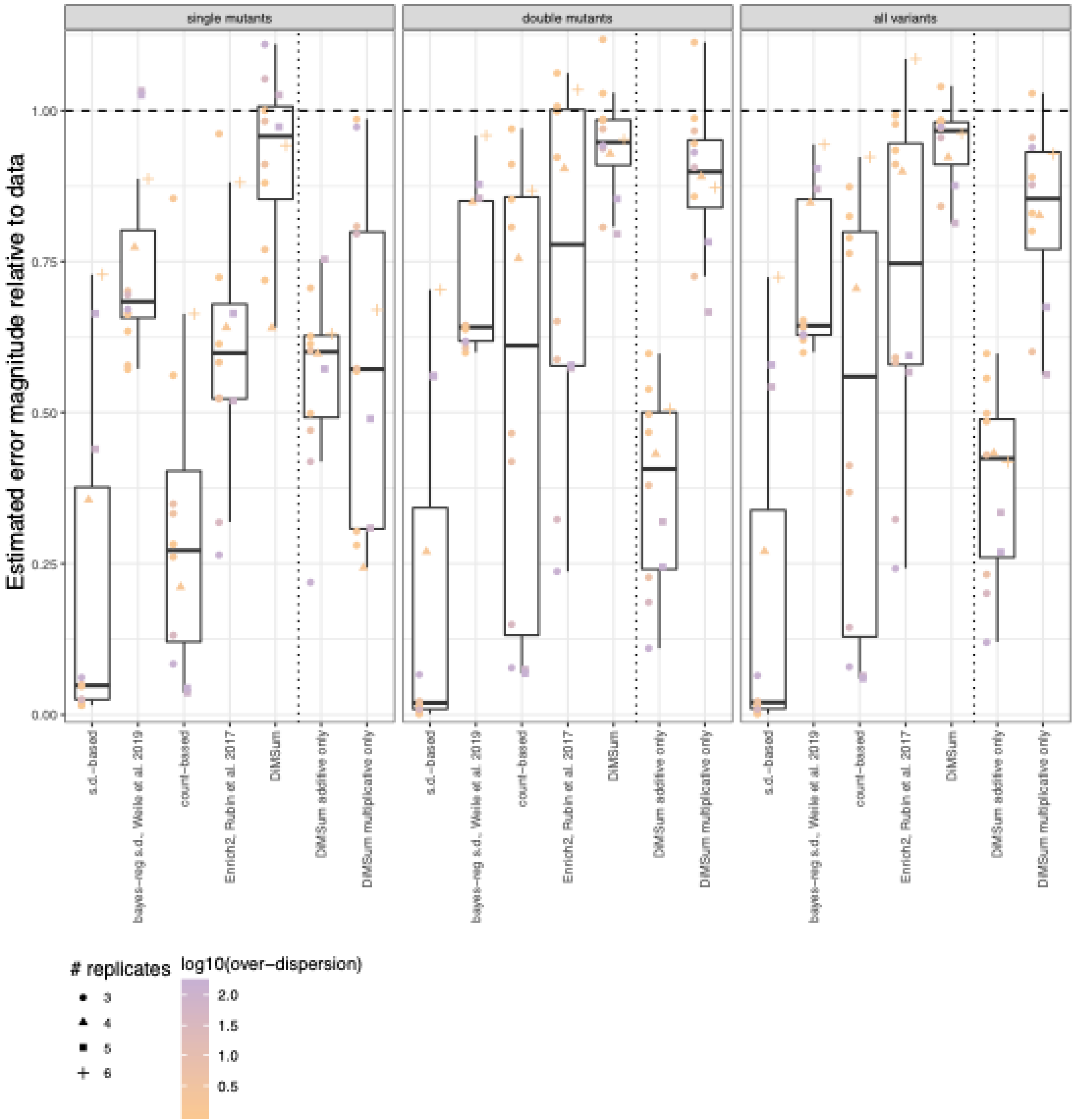

Figure S8. **Leave-one-out cross validation of different error models on twelve DMS datasets**. Leave-one-out cross validation of different error models on twelve DMS datasets stratified by single and double mutants and also showing performance of partial DiMSum error models. Color gives an estimate of the over-dispersion in the datasets (estimated as average over all multiplicative error terms of the DiMSum error model; yellow - litte, blue - a lot) to show dependency of the performance of count-based error models on over-dispersion. Point shape indicates the number of replicates in the dataset to show dependency of the performance of empirical variance based error models on the number of replicates. Single or double mutants are on nucleotide level (tRNA datasets) or amino acid level (all other datasets). 'DiMSum additive error' indicates DiMSum error model using only additive error terms (setting multiplicative terms to 1). 'DiMSum multiplicative error' indicates DiMSum error model using only multiplicative error terms (setting additive terms to 0).

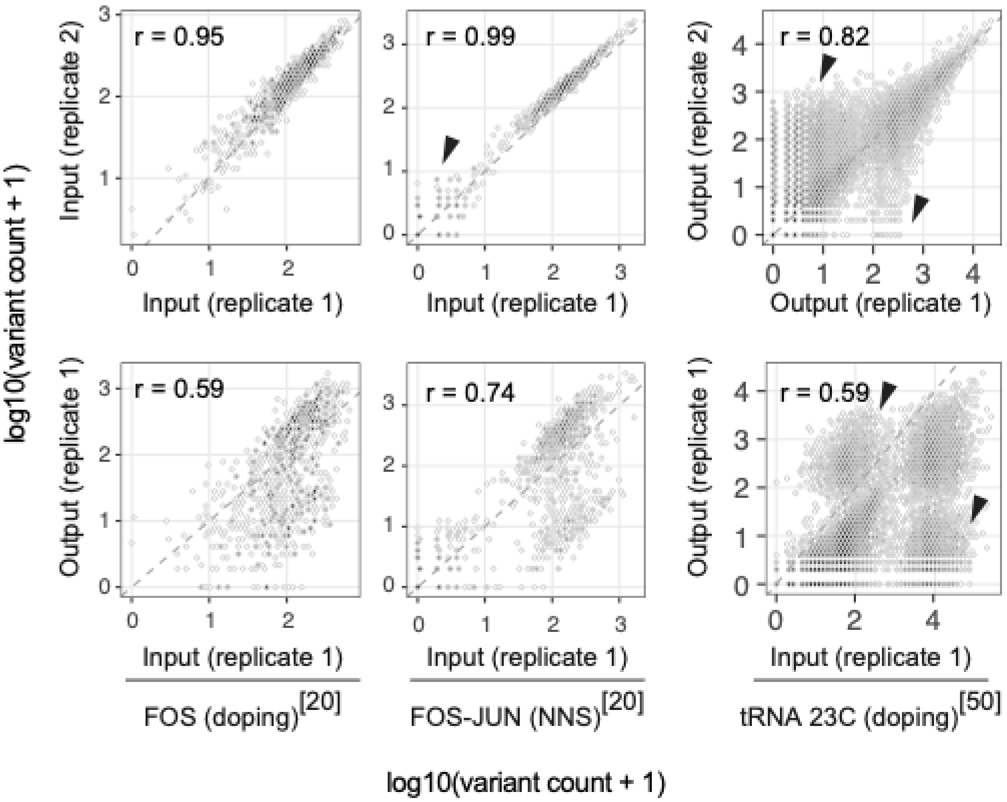

Figure S9. Scatterplots of Input and Output counts of previously published DMS experiments[20,50]. Only variants with two nucleotide substitutions are shown. For FOS and FOS-JUN datasets, counts of single AA variants with two nucleotide substitutions in the same codon are shown. The black arrows indicate populations of spurious double nucleotide variants originating from sequencing errors.

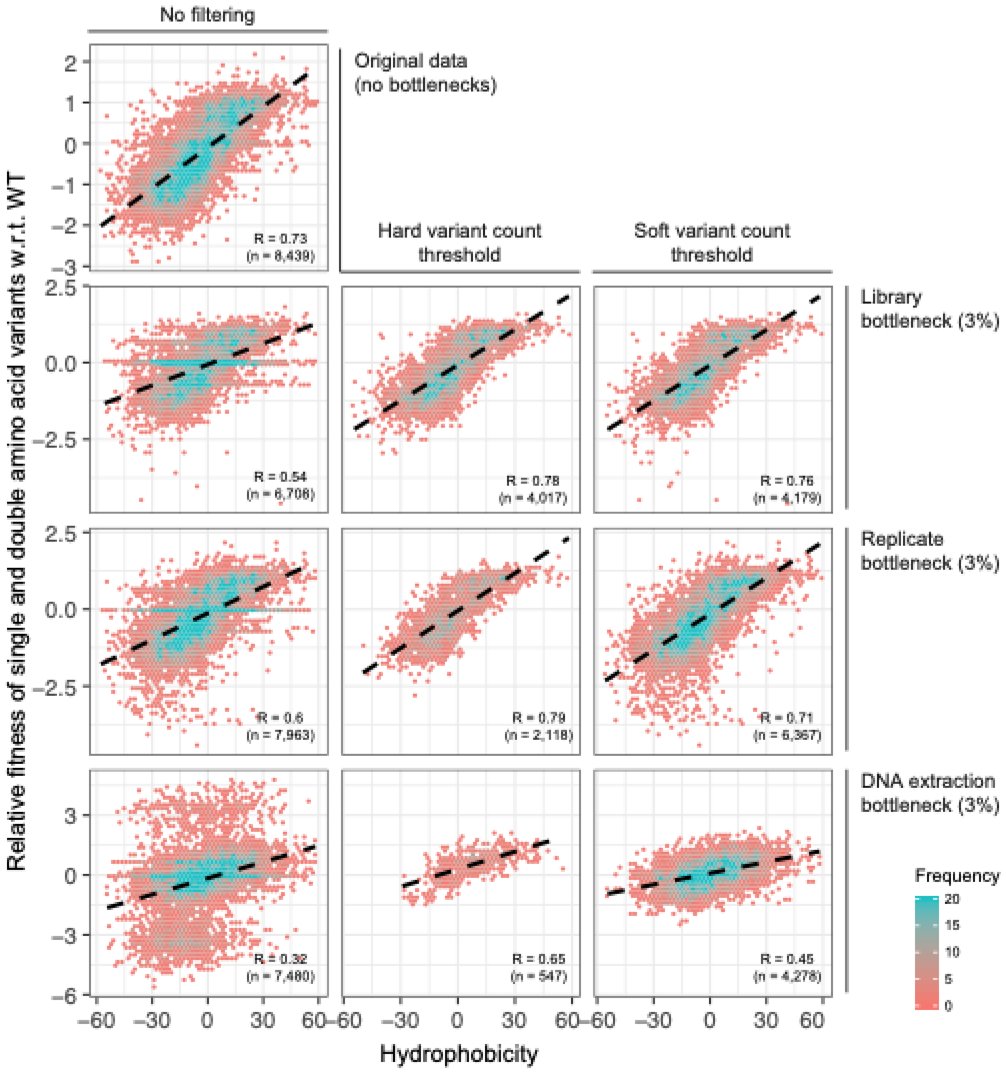

Figure S10. Effects of bottlenecks and variant filtering on fitness estimates and biological conclusions. Relative fitness of single and double amino acid variants as a function of hydrophobicity changes induced by mutation. Results are shown separately for the original data (no bottlenecks) as well as after simulating library, replicate and DNA extraction bottlenecks (3%). The middle column indicates the results after imposing a hard variant count threshold of 10 reads. Relative fitness was calculated from changes of output to input frequencies with respect to the WT sequence. The rightmost column indicates the results after imposing a soft variant count threshold of 10 reads (the original data was not filtered). For the DNA extraction bottleneck, read count thresholds were similarly applied to output samples.
