# Supplementary material for: DiMSum: an error model and pipeline for analyzing deep mutational scanning data and diagnosing common experimental pathologies
Source: Genome Biol. 2020 Aug 17;21:207. doi: 10.1186/s13059-020-02091-3 (PMC7429474; doi:10.1186/s13059-020-02091-3)
Supplement: Supplementary file 2 — Additional file 2. Review history. [file 13059_2020_2091_MOESM2_ESM.docx]

Review History

**First round of review**

**Reviewer 1**

**Are you able to assess all statistics in the manuscript, including the appropriateness of statistical tests used?**

Yes, and I have assessed the statistics in my report.

**Comments to author:**

(Signed review: Jesse Bloom)

This is a paper describing a very nice computational pipeline for the analysis of deep mutational scanning data. The approach implemented in the package nicely recognizes that statistical counting error on the sequencing often isn't the main source of experimental error.

The analysis of deep mutational scanning data is useful for a wide variety of purposes, and several different analysis packages (as well as many custom scripts) are in use. While it is unclear to me if any package will ever become the standard for this field because the analysis is so dependent on the specific details and goals of the experiment, this package certainly seems highly useful for some important classes of experiments and appears to offer some useful new approaches (as well as nice implementations of existing ones). Additionally, the authors should be commended for packaging the code they are using for their analyses and making it available to others.

I therefore support publication of this manuscript after requesting the following very manageable revisions:

1. (MANDATORY REVISIONS): The authors never give a theoretical statistical basis why experimental factors like bottlenecks in the selection of PCR should specifically cause the errors to increase in a multiplicative fashion from those calculated from counting statistics. While this mathematical form seems "reasonable" to me, I was left wondering where it came from. If there is a theoretical statistical basis why bottlenecks should give multiplicative error terms of the mathematical form described in the manuscript, then this should be derived and explained. If the multiplicative form is just a heuristic that empirically seems to capture bottleneck-like forms of error, then that is also OK---but in that case this should be clearly explained.

2. (HIGHLY DESIRABLE REVISION): The package is reasonably well documented on GitHub, but its usability would be dramatically increased for those outside the Lehner lab if there was an actual working example. The lab has performed DMS experiments and I'm guessing they have used this pipeline or ones similar to it to analyze their data. Can they include (on GitHub and linked in paper) an actual working example of running the analysis on a real data set, such as in a R Markdown notebook or a Jupyter notebook? Because of the lack of such a dataset, I wasn't able to run the pipeline myself in a meaningful way when reviewing the paper.

3. (MANDATORY REVISION): Better installation instructions would be good. In order to install DiMSum, I had to install the ShortRead package from Bioconductor, but it wasn't very clear that this needed to be done (or how) from the instructions. Also, could you make clearer that cutadapt, etc, need to be installed externally, and then the path set so that they are available under their names from the command line prompt.

4. (OPTIONAL REVISION): Related to the above, it would be really great to make a bioconda package that simply installs DiMSum and all of the dependencies.

**Reviewer 2**

**Are you able to assess all statistics in the manuscript, including the appropriateness of statistical tests used?**

Yes, and I have assessed the statistics in my report.

**Comments to author:**

The ms under consideration deals with processing of deep mutational scanning data. The authors propose a particular error model and integrate it into a processing pipeline. Much of the paper is devoted to describing particular artefacts in the data and discussing how they affect data and analysis.
Core of the manuscript is the error model which adds multiplicative error and an additive term to the model of Rubin et al (2017, their ref 36). Initially, in an attempt at understanding the shape of the variance function (sum of inverse counts), this reviewer had to go back and study ref 36. Admittedly, there no reason is provided either (they only refer to a book which over the holidays I didn't have access to) but it would have been nice of the present authors had they given some explanation why they use it in the first place. The present authors argue that certain experimental influences justify their modification to the error model. Throughout the paper they discuss these different artifacts.
The processing pipeline advertised in the title is really only of secondary importance in this manuscript and no comparison/evaluation of the pipeline as such is presented.
It would have been interesting to see some justification for the error model in terms of raw data. Instead, figures show only estimated error parameters, and not how the raw data would motivate the model. As it stands, the ms simply claims that these multiplicative and additive terms are needed. The fact that afterwards they can be estimated is not a justification why they were needed. Likewise, adding exactly these error terms to data in order to later on re-identify them does not prove that this is the right description of the data in the first place. Furthermore, it is not clear how different the present approach really is from the Rubin et al mixed-effect model. See below.
Some phenomena in the figures would merit explanation. For example, 3 is a key figure for the paper, but this reviewer had great problems deciphering what is actually shown. It is intriguing that the scatter plot in 3A shows a weak cloud to the left of the major cloud of points. Why this bimodality? Does it have something to do with the bimodality postulated later for the sequencing errors?
Many sentences are very long and difficult to parse. Terminology is fuzzy. When do the authors refer to read-counts, when to "fitness", when to "relative fitness"?  Sometimes "(random) variability" is associated to read-counts, or should it be to fitness? Naming conventions in the paper are very hard to follow. Example: Is "uncertainty" the same as "variability", the same as "error"? Has either of these terms been defined? What exactly are "bottlenecks"? What is the "sequencing count-based Poissonian variability expectation" (see legend to Fig. 3)? How does this quantity relate to the axes in 3A, which are a "fitness error" on the x-axis and a "variance" on the y-axis? Formulae are often integrated into the text and only readable on the screen with large zoom.
Assumptions are not made transparent. Is the "Poissonian" in "Poissonian read-counts" a model assumption? Probably yes, but it should be made explicit. After modeling of the data, claims are made about replicate scores being normally distributed. Has this been shown?
The present paper builds heavily on Rubin et al and the necessity of the modification introduced is not proven properly. The key section is "Error model validation and benchmarking". There, the present model is compared to three other ones, with the Rubin et al model the third. The prior two really are not state-of-the-art anyway. Then comes the challenge of showing superiority over the mixed-effect model of Rubin et al. However, instead of providing hard facts the authors simply repeat that their model is better because Rubin et al values "are only estimated from n replicates and therefore still underestimate true variability". No further evidence provided.
Thus, the present ms suffers from a number of shortcomings. It is not clearly written and in large parts it is really confusing. The error model is not justified sufficiently, but instead the data get studied only from the point of view of this error model. It is not shown that an actual advance has been achieved over Rubin et al. The advertised pipeline is described but no comparison is given to other available pipelines. The Results Section deals to a large extent with the experimental artefacts influencing the data, where the discussion is very vague and full of ill-defined terminology.

**Reviewer 3**

**Are you able to assess all statistics in the manuscript, including the appropriateness of statistical tests used?**

Yes, and I have assessed the statistics in my report.

**Comments to author:**

The manuscript introduces DiMSum, a software package for processing raw Deep Mutational Scanning (DMS) data to calculate variant effects and their associated measurement uncertainty. The software also produces a number of quality control outputs that help in detecting an array of sources of error. The authors evaluate the software on a number of existing DMS datasets as well as modified data with artificially increased error. The authors then discuss the different possible sources of random and systematic error and provide recommendations as to their detection and minimization. Finally, the authors demonstrate how different types and amounts of error affect biological conclusions drawn from existing data. Overall, the paper is well-written and the tool described within introduces a number of improvements over existing methods, in particular the ability to attribute different sources of error. There are only a few issues that need to be addressed, and they are entirely addressable.

Major comments:
1.     It is clear that the pipeline has features unavailable in other pipelines for analyzing DMS data.  However,  given the dozens of publications of DMS maps, at least several of which provided error estimates, there should be a clearer explication of what is new and what is not.   A fuller set of citations of previous analysis methods, with a 'feature table' would be an essential guide to the reader.
2.     It is concluded that DiMSum's error estimation is "outperforming previously used methods", it should be made clear that the comparison here was with other methods of the authors' own devising, and that this comparison was NOT made against all previous analysis approaches.  Although it would be nice to have a head-to-head comparison between this and all previous methods, the dataset may not yet exist to do this properly, and the authors should be let off the hook for this.  However, the authors should make clear that there are methods that have NOT been tried in this analysis.  For example, a quick scan of recent DMS papers turned up one that used a Bayesian approach to error estimation that combined an error model similar to one used here with the observed differences between replicates (PMID 29269382).  How is this method different than Enrich (PMID: 28784151), which is probably the most popular current approach?
3.     As stated (both in the main text and in the methods section) that to calculate fitness scores, the authors calculate the log ratios between the raw read counts but do not clearly describe normalizing these counts with respect to the total number of reads in a given sample (in terms of replicate, conditon, etc). As sequencing depth can vary considerably between samples this could potentially introduce substantial systematic error. The authors clearly are aware of this and mention a "simple scale and shift procedure to minimize inter-replicate differences" in the Results section (page 9, line 11),  but there was no description of this in the Methods section. Even if such a replicate normalization is performed it is unclear how well this can correct for substantial differences in sequencing depth between conditions.
4.     The authors describe their error model as being based on the assumption of read counts following a Poisson distribution. Based on this description I was unable to follow how they arrived at the formula they show (page 17, line 19). Since the mean and variance of a Poisson distribution both correspond to lambda, the variance associated with N reads would also be N. Following these assumptions through propagation of uncertainty to derive the variance for the log ratio between N_output and N_input did not lead me to the equation the authors show. Please explain in more detail how this equation was derived.
5.     Figure 3C-D show that artificially increasing the multiplicative and additive error in one of the replicates is correctly reflected in the relevant model parameter. However, it would also be important to show that in each case the respective unrelated parameter remains relatively unchanged.
6.     It is not clear whether the software can handle input of a control 'wild-type' sequencing library, which enables (often sequencing instrument cycle-dependent) estimation and subtraction of base-calling errors.

Minor comments:
1.     The performance measure used in Figure 3B and Supplementary Figure 10, described as the "inverse of the standard deviation of the normalized (observed divided by predicted) fitness score variability between unseen and training replicates" is somewhat difficult to wrap one's head around. Only after going into the details of the Methods section did I feel like I understood what was being calculated. It may be more intuitive to instead use a more common quality metric, such as the RMSD between the predicted and actual errors. But at the least, the relevant explanation in the results section should be made more clear. This should be explained a bit better in the relevant parts of the result section as well.
2.     More detail and rationale should be provided for the process removing non-intended mutations.  If the mutations are really in the library, why would we not want to score them?
3.     Installation of the DiMSum R package is contingent on a fairly large number of software dependencies, only some of which are automatically installed with it. In terms of R packages, this is due to the fact that some of the dependencies are not available on CRAN, but via Bioconductor. Including a "biocViews:" tag in the package's DESCRIPTION file would solve that problem. (See https://github.com/r-lib/devtools/issues/700#issuecomment-235127291 ). In the case of external binaries, I found that DiMSum requires some of them to be renamed from their default, which was not documented. A CONFIGURE script would be helpful for the package build procedure to auto-detect the presence of the software under different names and to throw errors when the dependencies are not met. (See https://cran.r-project.org/doc/manuals/r-release/R-exts.html#Configure-and-cleanup )
4.     The manual / readme on the github page would benefit from some explicit examples / demos.
5.     Using the names 'input' and 'output' for the pre-selection and post-selection libraries can lead to some confusion, especially when talking about counts and scores, as these terms can be easily mistaken for meaning the input and output of the software or pipeline. A clearer alternative might be pre-selection and post-selection instead.
6.     The panels in Figure 3 should be re-ordered based on the order in which they are mentioned in the main text.
7.     The numbering of Supplementary Figures should follow their respective order in the main text.
8.     Since Figure 3B and Supplementary Figure 10 use the same metric, their axes should be labeled consistently.
9.     In Figure 3C, please describe in the caption what the labels 'input' and 'output' refer to exactly. If they refer to pre- and post-selection conditions (as opposed to the input and output of processing), then why does the error term in the post-selection condition drop in response to artificial increase of replicate 1 error?
10.     Better language could be used to distinguish (potentially-multi-variant alleles) from single nucleotide or amino acid variants.  E.g., "WRAP then tallies unique variant sequences, aggregates counts at the variant level"  It took some head-scratching to appreciate how tallying unique variant sequences was different from aggregating counts at the variant level"
11.     If the software outputs processed data in a format that is compatible with a current platform for DMS data deposition, e,g, MaveDB, this should be stated. If not, the authors may wish to consider adding this option.
12.     "low-order nucleotide variant" should be defined.

**Authors Response**

**Point-by-point responses to the reviewers’ comments:**

Reviewer #1: (Signed review: Jesse Bloom)

This is a paper describing a very nice computational pipeline for the analysis of deep mutational scanning data. The approach implemented in the package nicely recognizes that statistical counting error on the sequencing often isn't the main source of experimental error.

The analysis of deep mutational scanning data is useful for a wide variety of purposes, and several different analysis packages (as well as many custom scripts) are in use. While it is unclear to me if any package will ever become the standard for this field because the analysis is so dependent on the specific details and goals of the experiment, this package certainly seems highly useful for some important classes of experiments and appears to offer some useful new approaches (as well as nice implementations of existing ones). Additionally, the authors should be commended for packaging the code they are using for their analyses and making it available to others.

*Thank you very much for this assessment. We have now further improved and modularised DiMSum to increase its flexibility so that e.g. the error model can be run even if the preprocessing has been done independently: https://github.com/lehner-lab/DiMSum*

I therefore support publication of this manuscript after requesting the following very manageable revisions:

1. (MANDATORY REVISIONS): The authors never give a theoretical statistical basis why experimental factors like bottlenecks in the selection of PCR should specifically cause the errors to increase in a multiplicative fashion from those calculated from counting statistics. While this mathematical form seems "reasonable" to me, I was left wondering where it came from. If there is a theoretical statistical basis why bottlenecks should give multiplicative error terms of the mathematical form described in the manuscript, then this should be derived and explained. If the multiplicative form is just a heuristic that empirically seems to capture bottleneck-like forms of error, then that is also OK---but in that case this should be clearly explained.

*The multiplicative + additive form of our error model was derived as a heuristic that we noted to well describe the dispersion observed in many DMS datasets. We thus converged on the same error model form that has been used to describe errors in transcriptome datasets (e.g. in the popular DESeq2 package by Love et al., Genome Biology, 2014). Intuitively, the multiplicative errors are rooted in the idea that constrictions (at bottlenecks) or expansions (growth of assay cell population, PCR amplification) of the variant library size/complexity affect all variant counts by the same relative (multiplicative) factor (+- random sampling differences). Additive terms derive from the additivity of variances of independent random processes, with the additive terms constituting random processes in the workflow that are independent of variant frequencies.*

2. (HIGHLY DESIRABLE REVISION): The package is reasonably well documented on GitHub, but its usability would be dramatically increased for those outside the Lehner lab if there was an actual working example. The lab has performed DMS experiments and I'm guessing they have used this pipeline or ones similar to it to analyze their data. Can they include (on GitHub and linked in paper) an actual working example of running the analysis on a real data set, such as in a R Markdown notebook or a Jupyter notebook? Because of the lack of such a dataset, I wasn't able to run the pipeline myself in a meaningful way when reviewing the paper.

*DiMSum now includes a Demo mode and Demo datasets that can be used to quickly test the functionality of both WRAP+STEAM modules: https://github.com/lehner-lab/DiMSum/blob/master/docs/DEMO.md
DiMSum pipeline reports are now written in R markdown and include detailed explanations of all summary plots and tips to identify and deal with systematic errors in DMS workflows*

3. (MANDATORY REVISION): Better installation instructions would be good. In order to install DiMSum, I had to install the ShortRead package from Bioconductor, but it wasn't very clear that this needed to be done (or how) from the instructions. Also, could you make clearer that cutadapt, etc, need to be installed externally, and then the path set so that they are available under their names from the command line prompt.

*We have improved the DiMSum source code documentation on GitHub including better installation instructions and a clearer explanation of dependencies: https://github.com/lehner-lab/DiMSum/blob/master/docs/INSTALLATION.md
All packages (including those from Bioconductor) now install automatically
STEAM functionality (counts to fitness) now requires no external binaries to run apart from Pandoc (for R markdown reports).*

4. (OPTIONAL REVISION): Related to the above, it would be really great to make a bioconda package that simply installs DiMSum and all of the dependencies.

*We agree and are in the process of making a Bioconda package for DiMSum.*


Reviewer #2:
The ms under consideration deals with processing of deep mutational scanning data. The authors propose a particular error model and integrate it into a processing pipeline. Much of the paper is devoted to describing particular artefacts in the data and discussing how they affect data and analysis.
Core of the manuscript is the error model which adds multiplicative error and an additive term to the model of Rubin et al (2017, their ref 36). Initially, in an attempt at understanding the shape of the variance function (sum of inverse counts), this reviewer had to go back and study ref 36. Admittedly, there no reason is provided either (they only refer to a book which over the holidays I didn't have access to) but it would have been nice of the present authors had they given some explanation why they use it in the first place. The present authors argue that certain experimental influences justify their modification to the error model. Throughout the paper they discuss these different artifacts. The processing pipeline advertised in the title is really only of secondary importance in this manuscript and no comparison/evaluation of the pipeline as such is presented.

It would have been interesting to see some justification for the error model in terms of raw data. Instead, figures show only estimated error parameters, and not how the raw data would motivate the model. As it stands, the ms simply claims that these multiplicative and additive terms are needed. The fact that afterwards they can be estimated is not a justification why they were needed. Likewise, adding exactly these error terms to data in order to later on re-identify them does not prove that this is the right description of the data in the first place. Furthermore, it is not clear how different the present approach really is from the Rubin et al mixed-effect model. See below.

*Thank you very much for this detailed critique, which has helped us a lot to improve the manuscript.*

Some phenomena in the figures would merit explanation. For example, 3 is a key figure for the paper, but this reviewer had great problems deciphering what is actually shown. It is intriguing that the scatter plot in 3A shows a weak cloud to the left of the major cloud of points. Why this bimodality? Does it have something to do with the bimodality postulated later for the sequencing errors?

*(Note previous Figure 3 is now Figure 2) The bimodality of error estimates based on sequencing counts under Poisson assumptions results from the distribution of nucleotide mutants in the dataset (see Figure 1b). It contains relatively few single nucleotide mutants (378 single nucleotide mutants in a 126nt assayed sequence) that have high sequencing counts (on average: 3,466, thus low count-based error estimates, >> the weaker cloud to the left) but many more double nucleotide mutants (46,711 of all possible 69,741 double nucleotide mutants) with low sequencing counts (on average: 23, thus high count-based error estimates, >> the major cloud of points). We have added a description of this phenomenon in the figure legend and also describe this in the Background section of the manuscript.*

Many sentences are very long and difficult to parse. Terminology is fuzzy. When do the authors refer to read-counts, when to "fitness", when to "relative fitness"?
Sometimes "(random) variability" is associated to read-counts, or should it be to fitness?
Naming conventions in the paper are very hard to follow.
Example: Is "uncertainty" the same as "variability", the same as "error"? Has either of these terms been defined?
What exactly are "bottlenecks"?
What is the "sequencing count-based Poissonian variability expectation" (see legend to Fig. 3)? How does this quantity relate to the axes in 3A, which are a "fitness error" on the x-axis and a "variance" on the y-axis?

*We have attempted to simplify language and unify naming conventions in the revised manuscript.
Read counts are the number of sequencing reads in Input or Output samples that map to a specific variant. Fitness is the metric we calculate from Input and Output read counts to estimate the selective effects of variants relative to the wild-type variant.
Random variability arises in the DMS workflow (e.g. due to finite variant counts) and is thus a property of both the read counts as well as the fitness scores calculated from them.
We have cleaned up the confusion around uncertainty, variability and errors and now only use ‘(measurement) error’ throughout the manuscript.
We define bottlenecks generically as steps in which variant pool size is restricted, e.g. because too little DNA material was extracted from assay populations.
We have clarified the legends in Figure 2A (previously Figure 3A). The x-axis displays error estimates based on the base-line Poisson model (variance equals mean).*

Formulae are often integrated into the text and only readable on the screen with large zoom.

*We have increased the font size of equations and hope their readability has improved.*

Assumptions are not made transparent.
Is the "Poissonian" in "Poissonian read-counts" a model assumption? Probably yes, but it should be made explicit.

*Yes, that sequencing counts of a variant with a specific frequency in a sample follow a Poisson distribution is an assumption that goes back to initial attempts to model errors in RNA sequencing datasets (see e.g. Marioni et al., Genome Research 2008). We have now stated this explicitly as we introduce the error model in the Results section.*

After modeling of the data, claims are made about replicate scores being normally distributed. Has this been shown?

*Yes, this was previously hidden away in Supplementary Figure 10. We now show quantile-quantile plots of replicate fitness score differences against the theoretical expectation for a Normal distribution in Figure 3a, c.*

The present paper builds heavily on Rubin et al and the necessity of the modification introduced is not proven properly. The key section is "Error model validation and benchmarking". There, the present model is compared to three other ones, with the Rubin et al model the third. The prior two really are not state-of-the-art anyway. Then comes the challenge of showing superiority over the mixed-effect model of Rubin et al. However, instead of providing hard facts the authors simply repeat that their model is better because Rubin et al values "are only estimated from n replicates and therefore still underestimate true variability". No further evidence provided.

*Our model builds on the same base as the mixed-effect error model of Rubin et al., namely the assumption that sequencing read counts follow a Poisson distribution. Deviation from this, instead of fitting individual mixed-effect terms for each variant, we derive replicate specific modifier terms.
The two ‘standard’ error models, namely variance-based and count-based ‘Poisson’ models have often been used in studies analysing DMS experiments. We have now cited a representative sample of studies in the relevant Result section. We have also added a fourth alternative, a modification of the variance-based error model using Bayesian regularization introduced by Weile et al., Mol. Sys. Bio. 2017.
The hard facts that our error model more accurately estimates measurement errors in DMS datasets than the alternative error models were shown in Figure 3e of the manuscript. We have now expanded this into a stand-alone Figure 3, showing first how estimated error magnitude relates to observed differences between replicate fitness scores (panels a, c; and how the resulting P-value distributions would look like, panels b, d) for two example DMS datasets form our lab, and then the comparison of error model performance across 12 DMS datasets.*

Thus, the present ms suffers from a number of shortcomings. It is not clearly written and in large parts it is really confusing.

*We have attempted to improve the structure and clarity of the text.*

The error model is not justified sufficiently, but instead the data get studied only from the point of view of this error model. It is not shown that an actual advance has been achieved over Rubin et al.

*We have attempted to justify the form of our error model more clearly, also relating it to statistical models previously developed in the transcriptomics field (esp. DESeq 1&2 packages by Anders et al.). Moreover, the advance in accurately estimating measurement errors in DMS datasets over previous error models is clearly depicted in Figure 3 of our paper.*

The advertised pipeline is described but no comparison is given to other available pipelines.

*We have now included a table directly comparing DiMSum to other currently available methods (Table 1).*

The Results Section deals to a large extent with the experimental artefacts influencing the data, where the discussion is very vague and full of ill-defined terminology.

*We have attempted to improve the clarity of the section describing how variants might appear purely due to sequencing errors in the sequencing record due to bottlenecks in the DMS workflow. Moreover we now show examples of published DMS datasets that display such artefacts to emphasize the gravity of the issue.*

Reviewer #3:
The manuscript introduces DiMSum, a software package for processing raw Deep Mutational Scanning (DMS) data to calculate variant effects and their associated measurement uncertainty. The software also produces a number of quality control outputs that help in detecting an array of sources of error. The authors evaluate the software on a number of existing DMS datasets as well as modified data with artificially increased error. The authors then discuss the different possible sources of random and systematic error and provide recommendations as to their detection and minimization. Finally, the authors demonstrate how different types and amounts of error affect biological conclusions drawn from existing data. Overall, the paper is well-written and the tool described within introduces a number of improvements over existing methods, in particular the ability to attribute different sources of error. There are only a few issues that need to be addressed, and they are
entirely addressable.

*We thank the Reviewer for this positive assessment of our manuscript.*
Major comments:
1. It is clear that the pipeline has features unavailable in other pipelines for analyzing DMS data. However, given the dozens of publications of DMS maps, at least several of which provided error estimates, there should be a clearer explication of what is new and what is not. A fuller set of citations of previous analysis methods, with a 'feature table' would be an essential guide to the reader.
2. It is concluded that DiMSum's error estimation is "outperforming previously used methods", it should be made clear that the comparison here was with other methods of the authors' own devising, and that this comparison was NOT made against all previous analysis approaches. Although it would be nice to have a head-to-head comparison between this and all previous methods, the dataset may not yet exist to do this properly, and the authors should be let off the hook for this. However, the authors should make clear that there are methods that have NOT been tried in this analysis. For example, a quick scan of recent DMS papers turned up one that used a Bayesian approach to error estimation that combined an error model similar to one used here with the observed differences between replicates (PMID 29269382). How is this method different than Enrich (PMID: 28784151), which is probably the most popular current approach?

*We have added a new Table 1 describing the different statistical models that have been used to estimate measurement errors in DMS derived fitness scores.
Indeed, we didn’t perform an exhaustive comparison against all previous methods to calculate measurement errors on DMS data, not the least because many publications do use custom implementations and do not provide easy access to code. We do mention this now in the Result section ‘Error model benchmarking’
However, as far as we can tell, the most popular approaches in the past have been to either use the empirical variance of fitness scores across replicates to calculate measurement errors (and we have now added the Bayesian regularization approach by Weile et al. MSB2017 as well) or the sequencing counts based on the assumption that they follow a Poisson distribution. The methods by Weile et al. and Rubin et al. (Enrich2, which was one of the three alternatives we tested against in the first version of the manuscript) provide a crossover and use additional model parameters to improve error estimates, with the first merging a prior based on regression across sequencing counts of all variants with the empirical variance estimates of each variant (using three parameters for the regression); and the latter using the empirical variance as a mixed effect term where it exceeds the error estimate from the sequencing counts (using one parameter per variant, i.e. thousands per dataset).
Our approach differs in that it consequently assumes that all variants are affected equally by error sources that increase their measurement error over and above the Poisson expectation from their sequencing counts. Therefore we derive three additional parameters per experimental replicate to estimate multiplicative error sources in the input and output samples as well as additive error sources.*

3. As stated (both in the main text and in the methods section) that to calculate fitness scores, the authors calculate the log ratios between the raw read counts but do not clearly describe normalizing these counts with respect to the total number of reads in a given sample (in terms of replicate, conditon, etc). As sequencing depth can vary considerably between samples this could potentially introduce substantial systematic error. The authors clearly are aware of this and mention a "simple scale and shift procedure to minimize inter-replicate differences" in the Results section (page 9, line 11), but there was no description of this in the Methods section. Even if such a replicate normalization is performed it is unclear how well this can correct for substantial differences in sequencing depth between conditions.

*Sequencing depths can indeed introduce substantial differences in statistical power between replicates. Because the variance inferred from our model is based on the observed sequencing counts in each replicate (further modified by multiplicative and additive terms), it accounts for these differences in sequencing depth.
E.g. replicate 2 was sequenced twice as deep as replicate 1. A mutant variant is observed with 100 reads before selection and 50 reads after selection in replicate 1, but (due to doubled sequencing depth) with 200 reads before selection and 100 reads after selection in replicate 2. For simplicity let's assume multiplicative terms are 1 and additive terms are 0, the error for replicate 1 is thus sigma_1 = sqrt(1/100 + /50) = ~0,173, that for replicate 2 is sigma_2 = sqrt(1/200 + 1/100) = ~0,122. Their ratio is sigma_1/sigma_2 ~ sqrt(2), due to the doubled sequencing depth of replicate 2.
To calculate log-fitness scores, instead of normalizing by total counts per sample, we normalize the output / input count ratio of each variant by the output / input count ratio of the wild-type sequence. This has the effect of setting fitness of the wild-type variant to 0. If we were to additionally normalize each output / input count ratio by the total number of reads (or ratios thereof), this would cancel out because of the wild-type normalization. I.e. log-fitness = ([output_i / output_total] / [input_i / input_total]) * ([input_wt / input_total] / [output_wt / output_total]) = output_i / input_i * input_wt / output_wt.*

4. The authors describe their error model as being based on the assumption of read counts following a Poisson distribution. Based on this description I was unable to follow how they arrived at the formula they show (page 17, line 19). Since the mean and variance of a Poisson distribution both correspond to lambda, the variance associated with N reads would also be N. Following these assumptions through propagation of uncertainty to derive the variance for the log ratio between N_output and N_input did not lead me to the equation the authors show. Please explain in more detail how this equation was derived.

*This crucial step was unfortunately omitted in the first manuscript version. It is that the variance of a logged variable is the variance of the variable divided by the square of it’s expectation value: 2(log(x)) = 2(x)E[x]2 =1x. Therefore, for the calculation of measurement errors in fitness scores of a variant (note that we omit inclusion of counts from wild-type variant used for normalization, as these terms are negligible):
2(f) =2(log(NoutputNinput))=2(log(Noutput) - log(Ninput))= 2(Noutput)E[Noutput]2+ 2(Ninput)E[Ninput]2 =1Noutput+1Ninput*

5. Figure 3C-D show that artificially increasing the multiplicative and additive error in one of the replicates is correctly reflected in the relevant model parameter. However, it would also be important to show that in each case the respective unrelated parameter remains relatively unchanged.

*We have now split off the panels showing effects of artificially manipulating error terms to Supplementary Figure 7 to improve clarity of the main figure.
In Supplementary Figure 7 we now also show how the unrelated parameters change. We find that manipulating multiplicative errors have little effect on additive error terms. However, increasing additive error does change multiplicative errors. This is likely because the magnitude of manipulation is very large (1 s.d.-value, biggest observed additive errors are ~0.3 in real DMS datasets). At this magnitude, additive errors dominate the overall error, thus impeding reliable estimation of multiplicative error terms.*

6. It is not clear whether the software can handle input of a control 'wild-type' sequencing library, which enables (often sequencing instrument cycle-dependent) estimation and subtraction of base-calling errors.

*DiMSum currently does not handle this but we will consider adding this functionality in future updates.*

Minor comments:
1. The performance measure used in Figure 3B and Supplementary Figure 10, described as the "inverse of the standard deviation of the normalized (observed divided by predicted) fitness score variability between unseen and training replicates" is somewhat difficult to wrap one's head around. Only after going into the details of the Methods section did I feel like I understood what was being calculated. It may be more intuitive to instead use a more common quality metric, such as the RMSD between the predicted and actual errors. But at the least, the relevant explanation in the results section should be made more clear. This should be explained a bit better in the relevant parts of the result section as well.

*We have attempted to clarify the metric we chose to calculate error model performance. We have added a schematic to the top of figure 3 to explain better what is calculated in the leave one out cross validation: The difference in fitness scores between the merged score across training replicates ftrain and the ‘raw’ score of the test replicate ftest, normalized by the error estimates of both scores which are calculated from the error model that was trained on the training replicates. This z-score distribution is then shown in a quantile-quantile plot against the expectation of a Normal distribution (Figure 3a, c). As an alternative quantification we also plot the P-values resulting from a z-test of these z-scores as a quantile-quantile plot against the expectation of a uniform distribution (Figure 3b, d). Finally, we calculate the standard deviation across the z-score distribution to quantify whether error models estimate the correct error magnitude across 12 published datasets. The expectation here is that if error magnitudes are estimated correctly, z-score standard deviation should be one (it follows a Normal distribution with unit standard deviation). If the z-score distribution is wider, the error magnitude has been under-estimated, if it is narrow, the error magnitude has been overestimated. These estimates are given in Table 2. To facilitate visual comparison we plot the inverse of the z-score standard deviation in Figure 3e.*

2. More detail and rationale should be provided for the process removing non-intended mutations. If the mutations are really in the library, why would we not want to score them?

*Removing non-intended mutations is an optional feature in DiMSum and variants associated with non-intended mutations are in fact retained by default.
However, we assume that non-intended mutations are by and large sequencing errors, i.e. their fitness values are meaningless and will introduce systematic errors into down-stream analysis. We therefore recommend they be removed and provide this option which allows simple removal of these variants if so desired.*

3. Installation of the DiMSum R package is contingent on a fairly large number of software dependencies, only some of which are automatically installed with it. In terms of R packages, this is due to the fact that some of the dependencies are not available on CRAN, but via Bioconductor. Including a "biocViews:" tag in the package's DESCRIPTION file would solve that problem. (See https://github.com/r-lib/devtools/issues/700#issuecomment-235127291 ). In the case of external binaries, I found that DiMSum requires some of them to be renamed from their default, which was not documented. A CONFIGURE script would be helpful for the package build procedure to auto-detect the presence of the software under different names and to throw errors when the dependencies are not met. (See https://cran.r-project.org/doc/manuals/r-release/R-exts.html#Configure-and-cleanup )

*We thank the reviewer for these helpful tips. We have included the “biocViews” tag so that all packages (including Bioconductor) now install automatically
STEAM functionality (counts to fitness) now requires no external binaries to run apart from Pandoc (for R markdown reports). DiMSum itself auto-detects the presence of required software and throws errors when the dependencies are not met*

4. The manual / readme on the github page would benefit from some explicit examples / demos.

*DiMSum now includes a Demo mode and Demo datasets that can be used to quickly test the functionality of both WRAP+STEAM modules: https://github.com/lehner-lab/DiMSum/blob/master/docs/DEMO.md*

5. Using the names 'input' and 'output' for the pre-selection and post-selection libraries can lead to some confusion, especially when talking about counts and scores, as these terms can be easily mistaken for meaning the input and output of the software or pipeline. A clearer alternative might be pre-selection and post-selection instead.

*Thank you for this suggestion, but we prefer to stick with ‘Input’ and ‘Output’.*

6. The panels in Figure 3 should be re-ordered based on the order in which they are mentioned in the main text.

*This has been fixed, old Figure 3 is now Figure 2.*

7. The numbering of Supplementary Figures should follow their respective order in the main text.

*Fixed.*

8. Since Figure 3B and Supplementary Figure 10 use the same metric, their axes should be labeled consistently.

*Fixed.*

9. In Figure 3C, please describe in the caption what the labels 'input' and 'output' refer to exactly. If they refer to pre- and post-selection conditions (as opposed to the input and output of processing), then why does the error term in the post-selection condition drop in response to artificial increase of replicate 1 error?

*Yes, Input/Output does refer to pre/post selection conditions. See answer to related major comment 5. Input and Output multiplicative error terms have a certain overlap in how they affect variants (see insets in Figure 2a, b). In the manipulations shown in Supplementary Figure 7, reductions in Output 1 error terms are small compared to the 3-fold or 10-fold increase in Input 1 error terms.*

10. Better language could be used to distinguish (potentially-multi-variant alleles) from single nucleotide or amino acid variants. E.g., "WRAP then tallies unique variant sequences, aggregates counts at the variant level" It took some head-scratching to appreciate how tallying unique variant sequences was different from aggregating counts at the variant level"

*We have improved this to reduce confusion.*

11. If the software outputs processed data in a format that is compatible with a current platform for DMS data deposition, e,g, MaveDB, this should be stated. If not, the authors may wish to consider adding this option.

*DiMSum now outputs a MaveDB compatible .csv file with single nucleotide or amino acid variant fitness and error estimates by default*

12. "low-order nucleotide variant" should be defined.

*Fixed.*

**Second round of review**

**Reviewer 1**

The revised version satisfactorily addresses the major critiques, and is appropriate for publication.

**Reviewer 2**

This is a much improved manuscript now. Definitions are clear and the logical structure of the work is clean and easy to follow. Modeling decisions are well justified and the success of the approach is laid out convincingly.

**Reviewer 3**

The revision does a great job of improving an already-good manuscript.   The described methods promise to be very useful in the analysis of deep mutational scanning data
